# Immediate loading in partially edentulous patients with fixed implant-supported restorations cases report

**DOI:** 10.3389/froh.2024.1369494

**Published:** 2024-05-07

**Authors:** Shuang Wang, Siyi Duan, Rui Chen, Zijian Wang, Yulong Tang

**Affiliations:** ^1^ Graduate School, China Medical University, Shenyang, China; ^2^ Department of Stomatology, General Hospital of Northern Theater Command, Shenyang, China; ^3^ Graduate School, Dalian Medical University, Dalia, China

**Keywords:** multiple adjacent missing, esthetic zone, screw-fixed framework, osteotomy, immediate load, case report

## Abstract

**Objectives:**

This article reports on four rare cases involving multiple trauma-induced adjacent missing anterior teeth in the maxillary or mandibular region. These cases were successfully treated using a 4-axial implant-based alternative insert and an immediate loading protocol.

**Material and methods:**

This series of cases was summarized by retrospective study that 4 patients who received a total of 20 immediately loaded implants. These patients had suffered from trauma-induced loss of 8–9 adjacent anterior teeth. The 4-axial-implants were inserted with the assistance of digital pioneer drill guides. The surgical procedure involved alveolar bone trimming or ultrasonic osteotomy, eliminating the need for traditional large-area bone augmentation. Pre- and post-operative CBCT was matched using DTX Studio Implant software, the deviation of implant between actual position and preoperative design was measured and compared using SPSS software package.

**Results:**

The average follow-up duration 48 months after implant prostheses, the cumulative retention rate of the implants was 100%, the marginal bone loss averaged 0.53 mm (SD 0.15 mm), and buccal plate bone loss averaged 0.62 mm (SD 0.41 mm).

**Conclusions:**

This retrospective clinical report demonstrates the successful treatment of several patients with multiple adjacent maxillary or mandibular anterior teeth using four implant-supported screws to fix the frame and employing immediate loading. The approach resulted in long-term stable clinical outcomes. Moreover, the method not only shortens the period of edentulism but also facilitates easy disassembly, maintenance, and cleaning. Consequently, it emerges as a highly favorable clinical option for patients suffering from extensive tooth loss.

## 1 Introduction

The sixth ITI Treatment Guide offers comprehensive schemes for implant restoration of multiple adjacent missing teeth in the esthetic zone, offering clear recommendations for restoring 1–6 teeth (1). However, the ITI Treatment Guide does not specifically recommend an optimal number of implants for patients who have lost over 8 adjacent anterior teeth but are not completely edentulous (2). Traditionally, the treatment approach for these patients involves segmental implant restoration, synchronized or earlier bone augmentation, and delayed loading (3). Unfortunately, this approach is associated with complications, including suboptimal aesthetic outcomes, lengthy treatment periods, significant trauma, and challenges related to extensive bone grafting (4). Patients often experience vertical and/or horizontal loss of soft and hard tissues, particularly in the maxillary anterior region, which is aesthetically challenging to rehabilitate (5, 6). Achieving satisfactory aesthetics often requires repeated guided bone regeneration and connective soft tissue augmentation, which increase the risk of operational and aesthetic failures. Patients not only desire “functionally stable implants” but also seek esthetic and functional rehabilitations within a shorter treatment time (7). Using traditional bone grafting surgery or free inferior alveolar nerve grafting not only increases the surgical difficulty and prolongs the operation time but also adds to the patient's expenses (8). The fifth Consensus Statements of the International Team for Implantology (ITI) propose that while the primary goals in the anterior esthetic zone are to achieve optimal esthetics and long-term stability without complications, secondary goals should also be considered to save chairside time and costs, alleviate postoperative pain or discomfort, and shorten healing time (9). Taking inspiration from the concept of All-on-4®, a protocol has been developed: 4-axial-implant alternated insert using 3D printed surgical guides for patients who have lost 8–10 adjacent maxillary or mandibular anterior teeth (10). This approach is being called Part-on-4, avoids the need for bone grafting through osteotomy and utilizes one-piece screw-fixed frameworks for immediate loading (11, 12). The remaining natural molars serve as occlusion supports. Part-on-4 simplifies surgery, shortens the edentulous period, and reduces the risk of residual bonding agents. Clinical cases and long-term follow-up observations have demonstrated the favorable clinical outcomes of Part-on-4. In this article, the purpose of this case series report is to present this part on 4 clinical protocol and report anecdotally the outcome for patients treated this way.

## 2 Material and methods

### 2.1 Basic information of patients

Patient gender, age, date of operation, edentulous zone, implant site, implant information, osteotomy height, primary stability, and abutment selection were recorded for each patient (Table 1).

**Table 1:** Basic information of patients undergoing part-on-4.

No.		Patient 1				Patient 2				Patient 3				Patient 4				Patient 4			
Gender		Male				Male				Male				Male				Male			
Age (years)		25				28				18				38				38			
Date of operation		2016/05/17				2017/12/01				2018/12/18				2019/01/11				2019/01/11			
Edentulous zone		35–44				34–44				34–44				14–25				34–44			
Site of implant		44	42	33	35	44	42	32	34	44	42	32	34	14	11	22	25	44	42	32	34
Implant	Type	Nobel CC				Nobel PCC				Nobel PCC				Nobel CC				Nobel CC			
	Length (mm)	13	13	13	13	11.5	13	13	11.5	11.5	13	13	11.5	13	13	13	13	11.5	13	13	11.5
	Diameter (mm)	4.3				3.75				3.75				4.3	3.5	3.5	4.3	3.5			
Height of osteotomy (mm)		0	2.5	1.8	0	1	3.5	1.7	1	0	3	2	1	0	2	1.5	0	4	5.5	6.5	3.5
Initial stability (N.cm)		35	45	45	45	35	45	45	45	35	45	45	45	45	45	35	35	45	40	40	35
Abutment	Type	MUA Plus				MUA Plus				MUA Plus				On 1® Base				MUA Plus			
	Angle (°)	30	0	17	0	0				0				0				0			
	Height (mm)	2.5	3.5	3.5	3.5	2.5				2.5				2.5				3.5			

### 2.2 Materials and instruments

The following materials and instruments were used: KaVo oral cone beam computed tomography (CBCT) and orthopantomograph (Kavo Company, Germany), Nobel On 1® Base, healing caps, and Nobel implanter (Kavo Company, Germany), NobelReplace Conical Connection and NobelParallel Conical Connection implants, multi-unit abutment (MUA-Plus), Mectron PiezoSurgery® piezosurgery system (Mectron Company, Italy), and digital implanting operation guides (Color Cube, Tianjin, China).

Four patients with a loss of 8–10 adjacent maxillary or mandibular anterior teeth, who desired fixed restorations, presented to the Department of Stomatology, General Hospital of Northern Theater Command between 2016 and 2022. All four patients included in the study had multiple anterior teeth loss due to trauma. They were treated using a standardized approach, and both clinical and imaging data were complete. Follow-up visits were conducted as scheduled. We waived the requirement for obtaining informed consent from patients and obtained approval from the Ethics Committee. This decision was based on the nature of our clinical retrospective study, which solely utilized pre-existing clinical records without administering additional treatment or imposing any risk or breach of patient confidentiality. The study received Institutional Review Board approval from the General Hospital of Northern Theater Command (No. 2022050).

### 2.3 Surgical procedure

**Figure 1:**
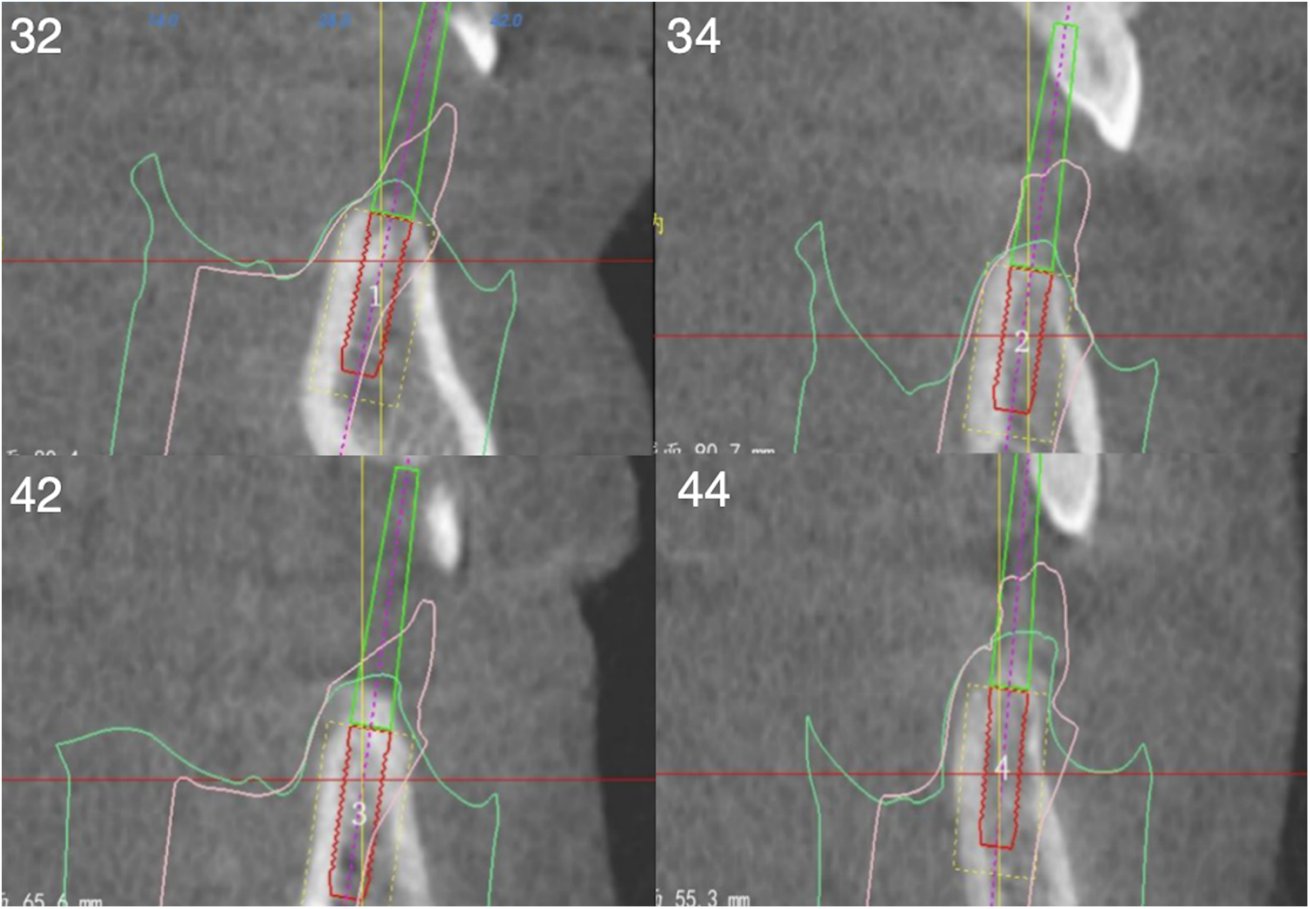
Preoperative CBCT: 32, 43, 42, 44 (FDA) measure the bone height of the implant site and simulate the placement of implants to evaluate the three-dimensional position.

**Figure 2:**
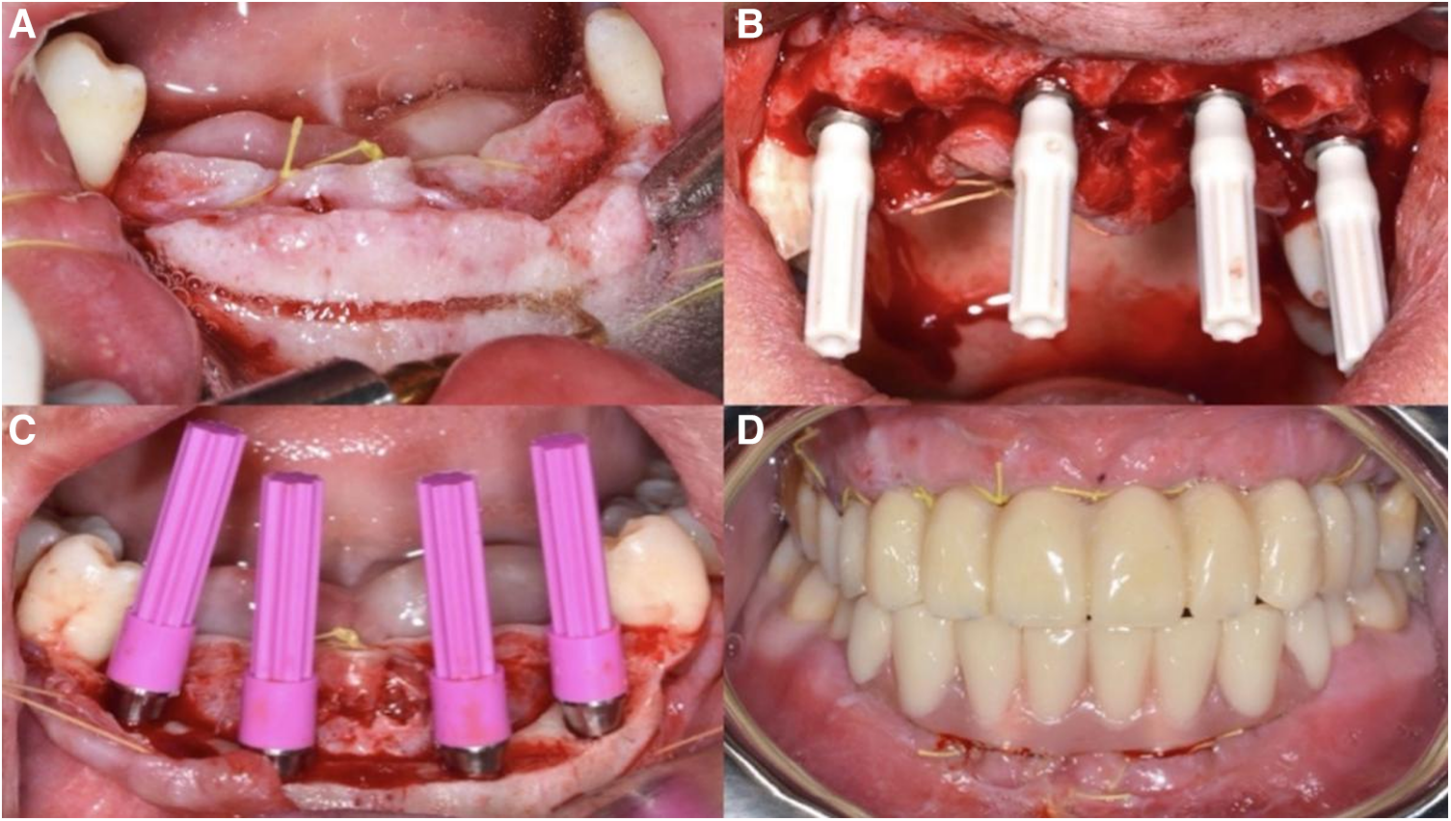
Surgical process images: (**A**) preoperative intraoral situation; (**B**) after all implants were implanted, the bone surface was leveled. (**C**) The Nobel MUA-Plus abutments have been screwed onto the implants; (**D**) make an immediate prosthesis and wear it in the mouth on the same day.

**Figure 3:**
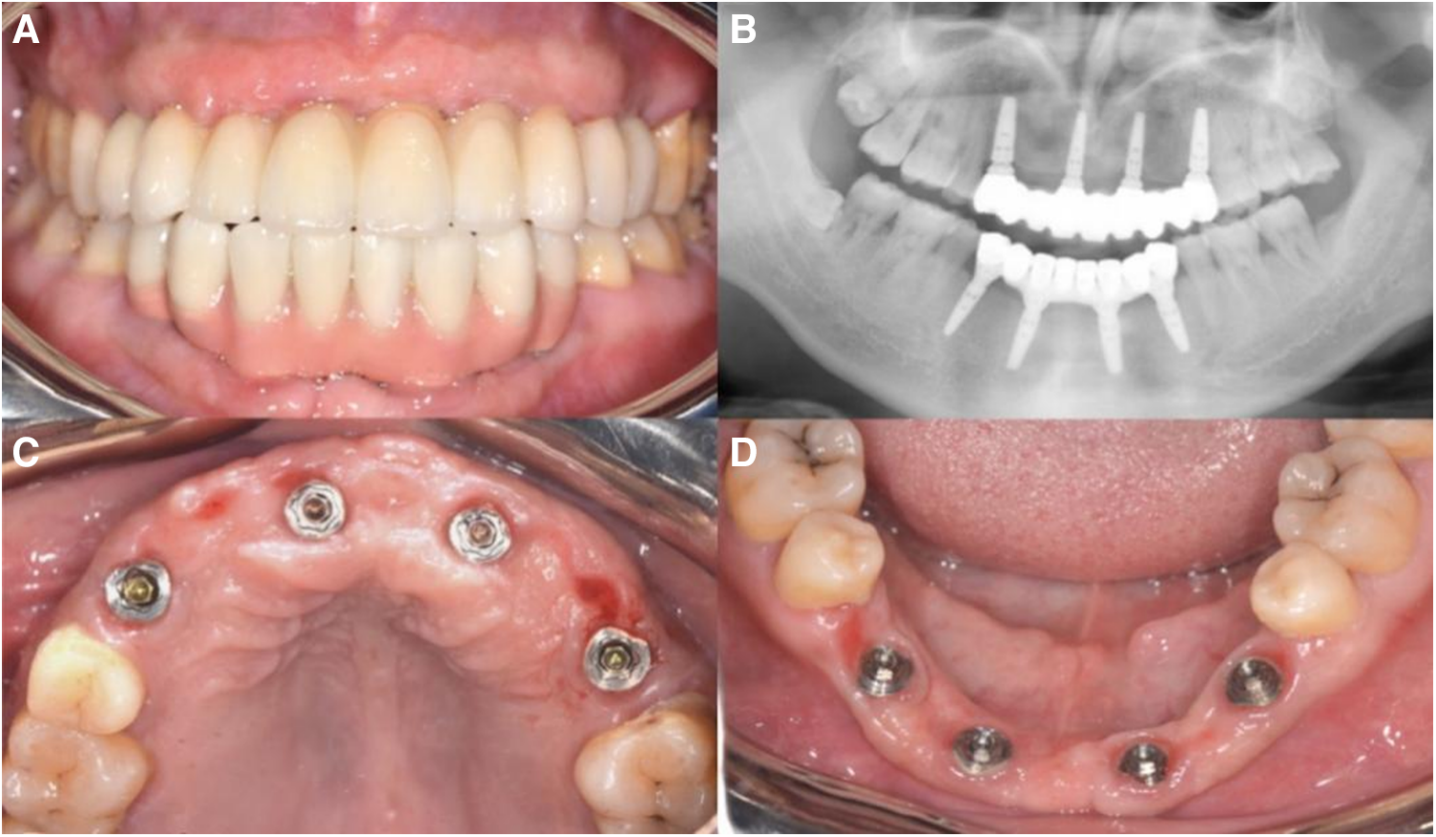
3-year follow-up images: (**A**) intraoral occlusal photograph: illustrating the occlusal relationship of the screw-fixed final restoration when worn in the patient's oral cavity; (**B**) the gingival tissue condition at 6 months postoperative during the final restoration; (**C**) photos of the occlusal surfaces inside the mouth after the placement of the final restoration, showing the emergence areas located either in the lingual sulcus or on the occlusal surface; (**D**) the curved cross-sectional image after the placement of the final restoration shows that the prosthesis is fully seated and the marginal bone around the implant is stable.

CBCT scans were taken to record the relevant data and determine the implant positions (Figure 1). A careful review of the patients' clinical and radiographic findings was conducted. Prior to implant placement, informed consent was obtained from each patient. 3D implant planning software (Nobel Clinician, Nobel Biocare USA) was used to optimize the placement of 4 implants in each arch based on the existing bone volume of each patient. Digital guide plates were designed and printed using 3Shape software (3Shape Company, Denmark). The evaluation included centric occlusion, esthetics, phonetics, and occlusal vertical dimension, with intraoral photos taken to document the state of the remaining teeth and soft tissues. Subsequently, a one-stage surgical approach was employed for the placement of all implants. The surgeries were performed under local anesthesia using Primacaine adrenaline 1/100,000 (Produits Dentaires Pierre Rolland, France). The bone surface was exposed by creating flaps, and for patients with insufficient alveolar crest width or unfavorable bone shape, ultrasonic osteotomy (4–6 mm) or high-speed drill bone trimming (0–3 mm) was performed. Digital surgical guides were used to prepare holes. Two implants with an insertion torque of 35–45 N/cm were placed in the anterior zone (maxillary central incisor or mandibular lateral incisor) and the premolar zone (first or second premolar) of each patient. For patients with sufficient maxillary bone volume, the bone surface below the framework was prepared in a fossa ovalis shape. Nobel MUA-Plus abutments or Nobel On 1® bases were then screwed on. After placing the healing caps, the soft tissues were readapted and sutured back into position using trans-gingival techniques. Postoperative CBCT scans were recorded to assess the accuracy of implantation site, buccal plate thicknesses, and distance from the inferior alveolar nerve canal. Temporary prostheses were fabricated using chairside impressions and worn on the same day, followed by an occlusion examination (Figure 2). Sutures were removed after one week, and clinical and radiographic examinations were conducted during the 3-month postoperative visit. Definitive impressions and face-bow transfer were performed, and temporary resin-cut frameworks were created. After try-in, the frameworks were confirmed to be accurate. The final prostheses, Ti-supported zirconia frameworks, were prepared and delivered 4–6 months after surgery. Subsequent follow-up visits with imaging and clinical periodontal examinations were scheduled annually (Figure 3).

### 2.4 Radiographic and clinical periodontal examination

**Figure 4:**
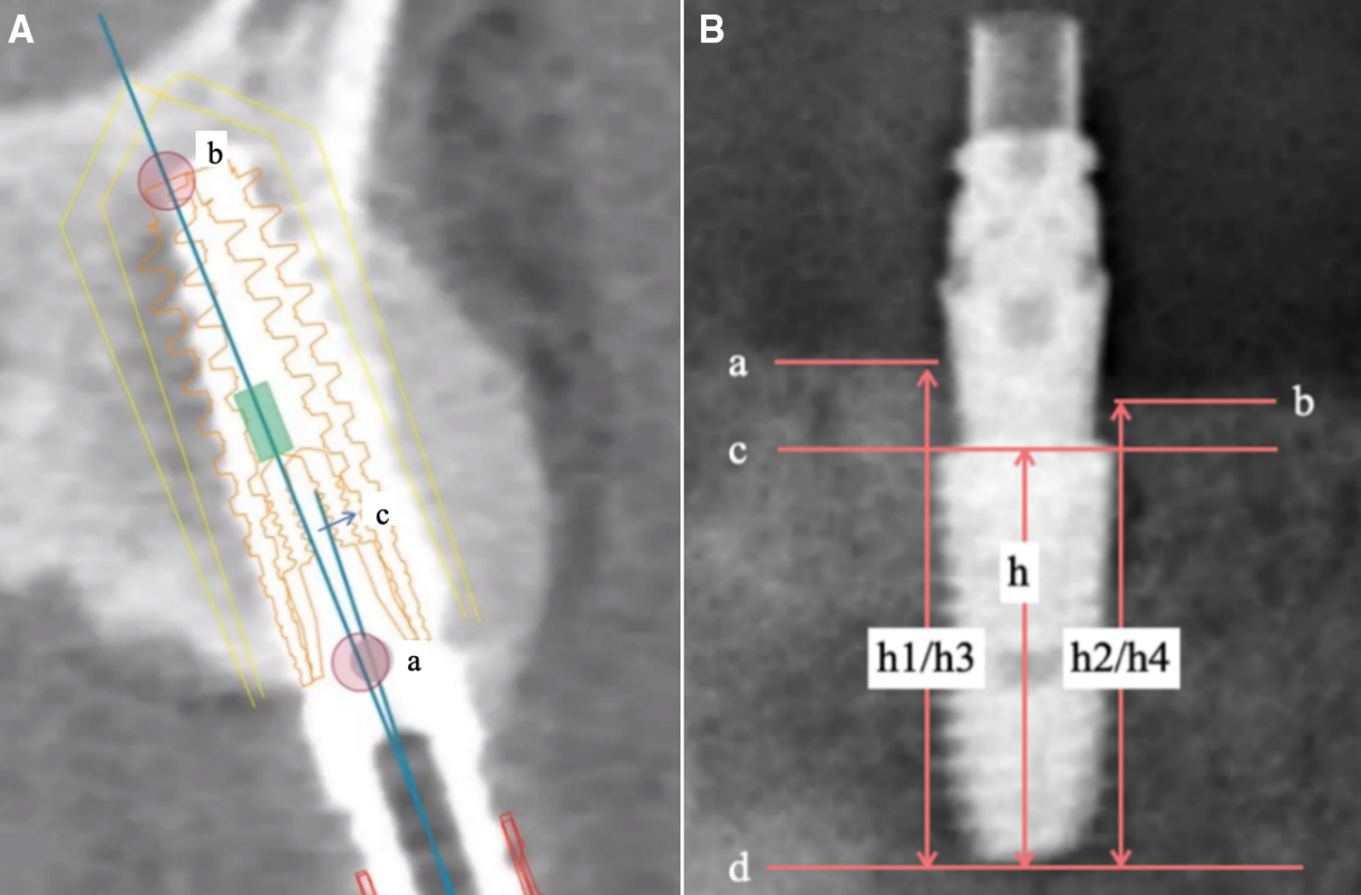
Data measurement: (**A**) DTX studio implant software deviation analysis: (a) implant platform (b) implant apex (c) implant angular; (**B**) measuring marginal bone resorption image using imageJ 1.48 software.

This case series report analyzes the mean deviation at the implant platform and implant apex, as well as the angular deviation, while utilizing digital pioneer drill guides. Pre- and post-operative CBCT was matched and integrated using DTX Studio Implant software, the deviation of implant between actual position and preoperative design was measured and compared using SPSS software package. During each follow-up visit, panoramic radiographs or CBCTs were performed to evaluate the positions of marginal bones and the thickness of the labial or buccal plate using ImageJ 1.48 software. The measured value is corrected according to the length of the inserted implants to avoid the error caused by the distortion of the image film. The bone level of each implant was measured at the proximal and distal points, and the difference between the bone level after operation and the bone level at the last follow-up was the bone loss at the edge of the implant during the follow-up period. All measurements were repeated three times a week by three trained surveyors, and self-consistency was tested (Figure 4). The measurements are calibrated with reference to the standard length (*h*) of the implant. The distances from the contact points between the implant and the alveolar bone to the plane of the implant root are denoted as h1, h2 immediately after the surgery, and h3, h4 at the last follow-up visit. The marginal bone loss (MBL) of the implant, denoted as Δh (measured in mm), at the follow-up time *t* (in years) is calculated using the formula: Δh = [(h1-h3) + (h2-h4)]/2. Furthermore, the peri-implant area was examined by doctors using a standard procedure to check for bleeding or suppuration. Peri-implantitis was defined as bleeding, suppuration, or bone resorption ≥2 mm in the peri-implant area discovered during probing examination, based on established criteria. Mechanical complications such as porcelain breakage, screw fracture, and prosthesis fracture were recorded annually. The gingival index was also recorded during the follow-up period, following the criteria set by Löe and Li et al. (13, 14). Finally, the survival rate and success rate of implants, marginal bone loss, and labial or buccal plate loss were calculated based on established criteria (15, 16). These measurements and evaluations provide important data to assess the clinical outcomes and effectiveness of the treatment approach.

## 3 Results

Each patient undergoes clinical and radiographic evaluations during follow-up visits at 3 months, 6 months, and 1 year post-surgery. According to the study findings, the mean deviation was as follows while using digital pioneer drill guides: the implant platform 1.28 mm ± 0.74 mm, apex 1.43 mm ± 0.75 mm, angular 4.21° ± 1.91°. During the follow-up of these 4 patients, none of the patients experienced pain, paresthesia, or other adverse symptoms the implant survival rate was 100%, comprising the absence of mobility, the absence of subjective complaints such as pain or paresthesia, the absence of peri-implant infection, and the absence of continuous radiolucency around the implant (17). The average gingival index was 0.28, indicating mild inflammation. That all implants remained in place and functional. Radiographic evaluations, including orthopantomography and CBCT, showed no significant alveolar bone resorption. The marginal bone loss averaged 0.53 mm (SD 0.15 mm), and buccal plate bone loss averaged 0.62 mm (SD 0.41 mm) (Table 2). These values indicate a stable peri-implant bone level. The implant success rate was also 100%, indicating successful integration and functional outcomes. During each reexamination, the prostheses were removed for examination and cleaning. However, no screw loosening, porcelain breakage, or fracture was observed. Minimal dental calculus and soft dirt accumulation were detected below the bridge, which were cleaned and polished. No abutment loosening was noticed. After each reexamination, the prostheses were reattached intraorally and manually screwed on. The patients' pronunciation, retroflexion, and plosive sound capabilities were examined, ensuring the restoration's functional outcomes. The patients were provided with information regarding oral hygiene maintenance and self-cleaning of the screw-fixed frameworks to ensure long-term success and durability of the restorations.

**Table 2:** Follow-up of patients undergoing part-on-4.

No.	Patient 1					Patient 2				Patient 3				Patient 4				Patient 4			
Final prosthesis	Ti-supported zirconia bridge																				
Months of follow-up	60 months					54 months				42 months				42 months				42 months			
Site of implanting	44		42	33	35	44	42	32	34	44	42	32	34	14	11	22	25	44	42	32	34
MBL (mm)	0.43					0.52				0.82				0.4				0.49			
Labial plate loss (mm)	0.17					1.36				0.7				0.39				0.5			
GI	0.31					0.33				0.38				0.13				0.25			

## 4 Discussion

### 4.1 Merits and demerits of part-on-4

Trauma-induced loss of consecutive teeth often leads to extensive horizontal and vertical bone defects. To address these bone deficiencies and determine the optimal site for guided implant placement, complex bone augmentation surgeries are typically required (18, 19). However, these surgical interventions can have drawbacks, such as compromising the aesthetic outcome due to mucosal scarring. The incidence rates of complications associated with these procedures were relatively high, a weighted mean gain of 8.04 mm and complications rate of 47.3% for distraction osteogenesis, 4.18 mm and 12.1% for guided bone regeneration (GBR) and 3.46 mm and 23.9% for bone blocks (20). Furthermore, a meta-analysis has shown that the implant success rate following complex bone augmentation procedures is only 75% during 1- to 5-year follow-up, indicating a relatively high risk of failure (21). This highlights the unpredictable clinical risks and potential complications associated with these bone augmentation surgeries (22). In summary, while various bone augmentation surgeries can achieve favorable clinical outcomes, the high incidence rates of complications and unpredictable risks of extensive bone grafting associated with operative wounds should be taken into consideration.

Our approach aimed to insert implants without bone subtraction or osteotomy in the maxillary anterior zone where are the esthetics crucial zone. The mandible anterior zone is not part of the esthetic zone, often with thin and blade-shaped alveolar ridge crest after tooth extraction. However, in cases with the sufficient bone height present, osteotomy may be a better choice for reconstructing bone width in the mandible anterior zone. Among the five cases, the osteotomy height was ≤3 mm in four cases and 4–7 mm in one case, the average osteotomy height was 2.03 mm, and the average bone width increment was 2.47 mm. Conversely, when the osteotomy height is suitable, the prosthesis/soft tissue junction (PSTJ) can be better hidden below the smile line, and the gingival margin and gingival papilla can exhibit more standardized shapes, without negative aesthetic effects (23).The osteotomy procedure is particularly suitable for patients with excessive gingival display, and the bone platform created after osteotomy should be positioned at least 4 mm below the smile line (5, 24). In cases where osteotomy was performed, we used Nobel MUA abutments without conducting gingival compression shaping due to insufficient keratinized gingiva. This method creates a natural gum line shape with fewer appointments. Osteotomy treatment resulted in great cosmetic outcomes and no loss of vertical bone tissue for Part-on-4 cases.

Additionally, this method is not entirely freehand implantation but involves designing a digital implant guide based on the oral structure of the patient and the surgical plan. The ideal three-dimensional position of the implants is accurately transferred to the surgical procedure using the guide, providing more precise operational guidance, including the positioning, angle, and depth of the implant socket (25). Immediate loading also reduces the edentulous period for patients, meeting their aesthetic and functional needs.

### 4.2 Indications of part-on-4

When a patient loses more than eight adjacent maxillary or mandibular anterior teeth, the number of available implant sites becomes restricted. In such cases, we recommend considering the following five points:

(1) Avoid the midline: The implant design should aim to position the two front implants as close to the lateral incisors as possible, avoiding placement along the midline. Eliminating cantilevers is necessary to ensure stability and support.
(2) Design the implant positions to allow for alternative, parallel, and symmetrical placement. This approach facilitates even distribution of horizontal stresses among the implants, enhancing overall stability.
(3) Utilize digitized guides to ensure accurate implant placement, achieve precise positioning, and minimize the margin for error.
(4) Optimal placement of prosthetic screw-access holes should be considered not only in the middle of the tooth tongue promontory but also between adjacent teeth when achieving mesiodistal positioning becomes challenging. This practice ensures appropriate access for maintenance and prevents complications.
(5) Factors such as patient age, gender, and living habits (e.g., overbite, overjet, smoking, neglect of oral hygiene) should be considered and addressed through oral hygiene education and maintenance guidance.

Additionally, the lower anterior zone surrounding the opening of the sublingual gland is susceptible to dental calculus accumulation, which presents a persistent challenge despite comprehensive oral hygiene education and maintenance guidance. This observation also applies to patients undergoing full-arch implant restoration. Screw-fixed bridges offer the advantage of being disassembled, cleaned, and maintained.

## 5 Conclusions

The Part-on-4 treatment offers significant advantages for patients with the loss of 8–9 consecutive maxillary or mandibular anterior teeth and stable posterior occlusion. It minimizes the duration of edentulism, enables regular disassembly, maintenance, and cleaning, making it a favorable clinical approach for this specific group of patients. Gingival shaping can enhance aesthetic outcomes when there is sufficient soft and hard tissue. In cases of inadequate bone volume, osteotomy serves as a viable alternative to bone grafting, reducing potential complications and wounds. Throughout the follow-up period, no substantial changes in labial bone plate thickness or marginal bone loss were observed, while the surrounding keratinized gum tissue remained healthy. However, it is essential to acknowledge that long-term clinical and aesthetic effects of the Part-on-4 treatment and osteotomy necessitate further research due to limited long-term observations and controlled studies. Continued research is crucial in evaluating the long-term efficacy, sustainability, and outcomes of Part-on-4 and osteotomy procedures for managing edentulous patients.

## Data Availability

The original contributions presented in the study are included in the article/Supplementary Material, further inquiries can be directed to the corresponding author.
